# Daily Scheduled High Fat Meals Moderately Entrain Behavioral Anticipatory Activity, Body Temperature, and Hypothalamic c-Fos Activation

**DOI:** 10.1371/journal.pone.0041161

**Published:** 2012-07-16

**Authors:** Christian M. Gallardo, Keith M. Gunapala, Oliver D. King, Andrew D. Steele

**Affiliations:** 1 Division of Biology, California Institute of Technology, Pasadena, California, United States of America; 2 Boston Biomedical Research Institute, Watertown, Massachusetts, United States of America; Florida State University, United States of America

## Abstract

When fed in restricted amounts, rodents show robust activity in the hours preceding expected meal delivery. This process, termed food anticipatory activity (FAA), is independent of the light-entrained clock, the suprachiasmatic nucleus, yet beyond this basic observation there is little agreement on the neuronal underpinnings of FAA. One complication in studying FAA using a calorie restriction model is that much of the brain is activated in response to this strong hunger signal. Thus, daily timed access to palatable meals in the presence of continuous access to standard chow has been employed as a model to study FAA in rats. In order to exploit the extensive genetic resources available in the murine system we extended this model to mice, which will anticipate rodent high fat diet but not chocolate or other sweet daily meals (Hsu, Patton, Mistlberger, and Steele; 2010, *PLoS ONE* e12903). In this study we test additional fatty meals, including peanut butter and cheese, both of which induced modest FAA. Measurement of core body temperature revealed a moderate preprandial increase in temperature in mice fed high fat diet but entrainment due to handling complicated interpretation of these results. Finally, we examined activation patterns of neurons by immunostaining for the immediate early gene c-Fos and observed a modest amount of entrainment of gene expression in the hypothalamus of mice fed a daily fatty palatable meal.

## Introduction

The ability of animals to time intervals to match resource availability is widely conserved in nature. Notably, light-entrained circadian behaviors related to sleep-wake cycles have a defined neural substrate known as the suprachiasmatic nucleus (SCN) of the hypothalamus [1]. In rodents, other circadian-like behaviors have been elicited by restricted feeding (RF) and calorie restriction (CR) paradigms, in which a meal is presented in limited amounts at a fixed time each day, usually in the middle of the light period [2], [3], [4]. Seminal studies have shown that after a few days, rodents start showing bouts of hyperactivity in anticipation of the scheduled meal, a behavior termed Food Anticipatory Activity (FAA) [5].

Given the circadian-like entrainment of FAA, initial studies attempted to determine the role played by the SCN in food entrainment, but found that FAA is not abolished by the ablation of this structure [6], [7], [8], [9]. In addition, since FAA persists beyond one cycle of total food deprivation at its original interval, researchers postulate the existence of a food entrainable oscillator (FEO) that is capable of operating autonomously at circadian intervals [3], [5]. Restricted feeding schedules result in an extreme catabolic state; importantly, starvation induces hyperactivity in rodents (especially mice), potentially confounding the behavioral readout of FAA.

Pioneering studies by Mistlberger and Rusak have shown that chronic food deprivation is not needed for induction of FAA, as free feeding rats fed a nutrient-rich palatable meal (PM) will show behavioral anticipation after several days on this schedule [10]. These initial results have been replicated and extended by examining entrainment of the immediate early gene expression of c-Fos in reward regions of the rat brain [11]. This PM paradigm is robust enough to change the phases of the Period family of clock genes in the brain [12]. Surprisingly, daily, timed chocolate treats induced changes in per1 expression in corticolimbic structures that remained in phase for at least 8 cycles after cessation of PM delivery in rats [12].

We determined recently that daily, timed PM can elicit moderate FAA in mice, opening up the possibility of exploiting the genetic resources available in this system [13]. Given the small size and high metabolism of mice, as opposed to rats, PM-elicited FAA in mice provides a viable alternative to using extreme hunger that can be induced by RF or CR. We use an automated, video-based behavioral recognition system to detect various activity behaviors in free-feeding mice [14]. Prior studies have shown the dependency of the PM paradigm on meal size and nutrient content [10]; therefore, we sought to determine the PM that would maximize anticipatory activity in mice. Based on empirical evidence from our previous studies [13] and limited pilot studies, we determined that fatty foods, as opposed to sweet foods (chocolate or “fruity”), had the greatest potential in maximizing this behavioral signature. As such we tested other fatty meals, including peanut butter, cheese, and rodent high fat diet for their potential to elicit FAA in mice. In addition, we measured core body temperature as an alternative readout of bodily rhythms to determine the extent of its entrainment to daily high fat or cheese PMs. Finally, we examined neural activation patterns at different times prior to PM delivery by immunostaining for the immediate early gene, c-Fos. We observed different patterns of c-Fos expression in anticipation of the daily high fat meal in the lateral hypothalamus and in the SCN. As all of these experiments were conducted in C57BL6/J strain and behavioral anticipation was never fully penetrant, we tested PMs on an additional strain of mice, 129S1, which had shown exceptional FAA to a CR feeding schedule, redistributing almost all activity to precede CR meal delivery. However, 129S1 mice fed daily high fat meals showed no behavioral anticipation of expected PM delivery.

## Results

### Experiment 1: Behavioral Responses to Palatable Meals of High Fat or Peanut Butter

**Figure 1:**
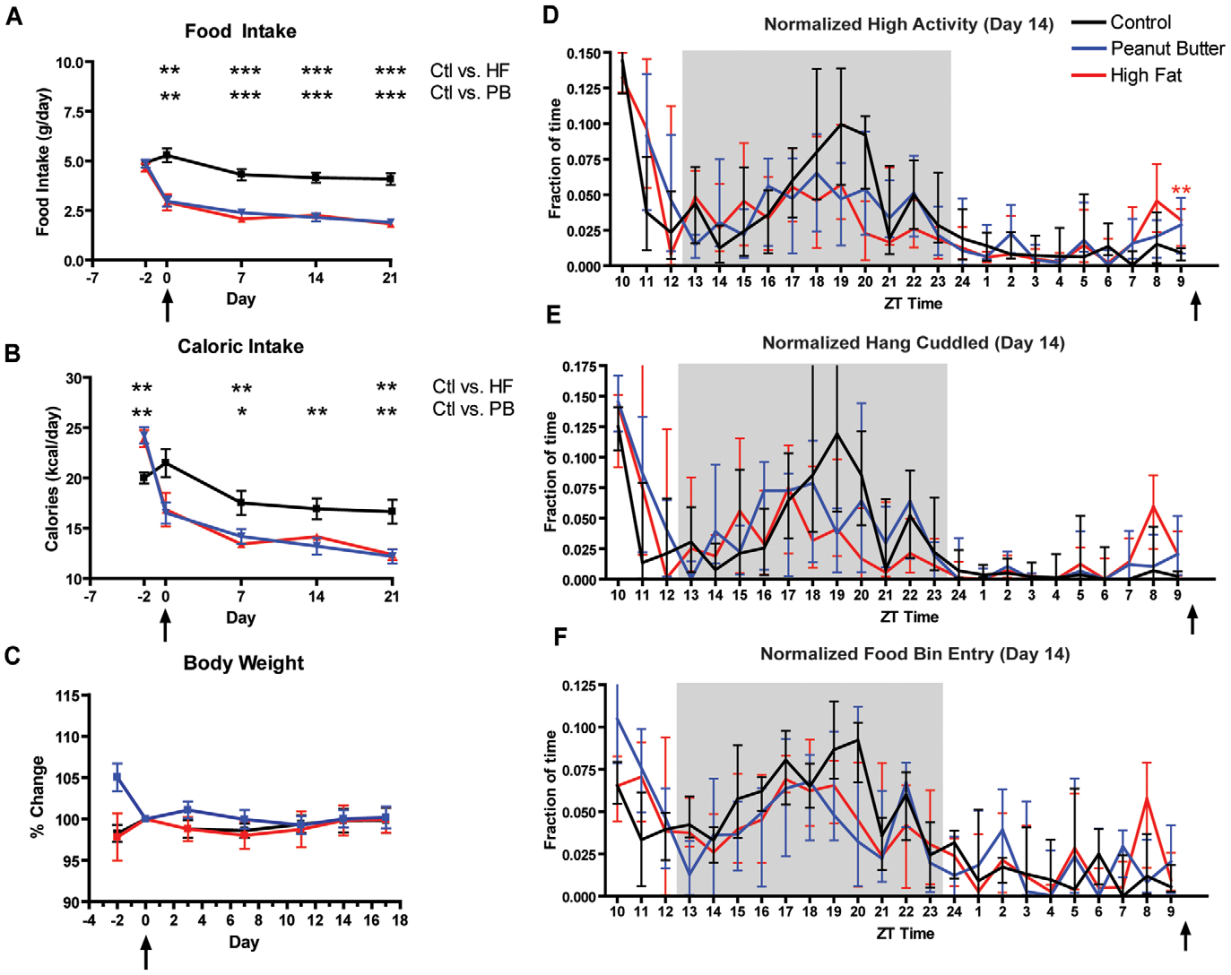
Food intake, caloric intake, change in body weight, and normalized activity profiles of mice in experiment 1. (A) Male mice fed peanut butter and high fat diet show a significant decrease in chow intake compared to mice with ad libitum access. The first measurement was made on day -2 (B) Male mice fed peanut butter and cheese show a significant 25% decrease in caloric intake (sum of calories from chow plus and PM) compared to mice with ad libitum access. Caloric intake values were estimated from nutritional facts provided by the respective companies. (C) Male mice in experimental groups do not show any significant change in body weight after 16 days of timed PM access, compared to ad libitum controls. (D) Normalized fraction of time per hour spent walking, hanging, jumping, or rearing observed during the 24-hour recording of Day 13 of experiment 1. Mice fed high fat show higher pre-prandial activity than those fed peanut butter, with fraction of high activity being significantly higher at ZT 9 compared to AL controls (E) Fraction of time per hour spent sniffing foo bin during 24-hour recording of Day 13. (F) Fraction of time per hour spent hang cuddling during the 24-hour recording of Day 13. Arrows after ZT 9 and below abscissa indicate time of PM. For 1A thru 1C, bars represent means ± SEM (significance was tested with Tukey-Kramer Multiple Comparisons Test with One-Way ANOVA post-test). For 1D thru 1F, bars represent medians+/-IQ Range (significance was tested with Mann-Whitney test with Dunn’s post test. * denotes p<0.05, ** denotes p<0.01 and *** denotes p<0.001.

In order to confirm and extend our initial finding that PMs are a suitable zeitgeber in C57BL/6J male mice, we designed a scheduled feeding study that includes AL access to food and daily-timed PM presentation of either rodent high fat diet (HF) or peanut butter (PB). Mice fed PB or HF ate their meal avidly, consuming it within 30 min or less throughout the study. Mice receiving a daily timed PM of PB or HF consumed significantly less chow than controls by their first day of scheduled feeding (p<0.01 for day 0 and p<0.001 from day 7 forward, Fig. 1A). Calories from both PMs, corresponding to 30–35% of their daily caloric intake, were calculated from their AL food intake preceding PM presentation. As previously observed [13], mice fed PMs of HF or PB had a significantly reduced overall caloric intake (the sum of calorie from the rodent chow plus the PM) relative to controls (Fig 1B). Surprisingly, this reduction in caloric intake did not manifest in changes in body weight between mice fed daily PMs and controls (Fig. 1C).

**Figure 2:**
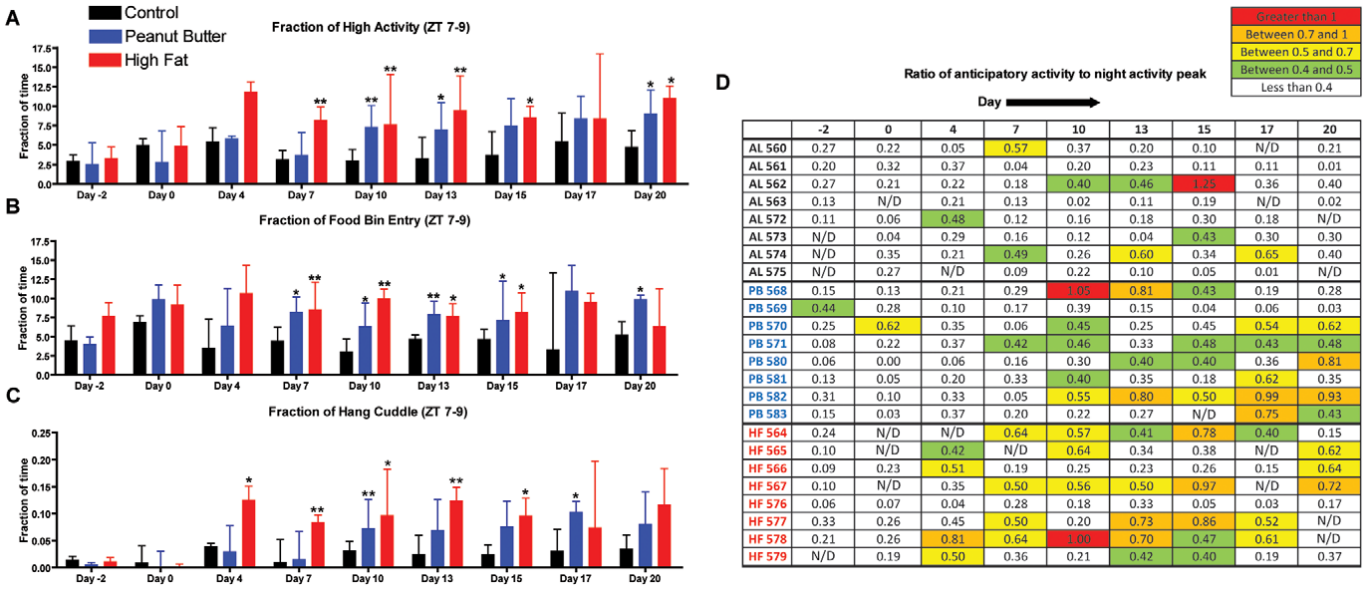
Home cage activity of mice in Experiment 1. (A) Fraction of time spent on high activity behaviors during ZT 7–9, the 2.5 h period preceding PM presentation (The sum of time of high activity behaviors during the 2.5 h before PM is divided by total seconds of high activity behaviors during entire recording period). (B) Fraction of time spent sniffing food bin (B) and hang cuddling (C) during the 2.5 h period preceding PM presentation. Mice fed high fat show overall an earlier onset of food anticipatory activity as measured by their significant increase in fraction of time spent in high activity and individual behaviors by day 7. This statistically significant trend persists to day 15. Mice fed peanut butter show a delayed onset of food anticipatory activity as measured by their high activity and individual behaviors, reaching statistical significance by day 10 and persisting until day 13. (D) The maximum number of seconds per hour of high activity behaviors in 2.5 h preceding mealtime is divided by the maximum number of seconds of high activity at nighttime. Ratios greater than 1 are shown in red, between 0.7 and 1 in orange, between 0.5 and 0.7 in yellow, and between 0.4 and 0.5 in green. Bars represent medians +/− IQ ranges. Statistics were performed using Mann-Whitney Test * denotes p<0.05 and ** denotes p<0.01.

Mice received their daily PM or were handled as a control at ZT 10 in a 13 h light: 11 h dark cycle (by convention ZT 12 is defined as “lights off”). Mice from all groups show a clear burst of activity (ZT 10 to ZT 12) after commencement of video recordings caused by their handling prior to the recording and their placement into new cages. Mice receiving daily timed HF exhibited a modest increase in the fraction of high activity (defined as the amount of time walking, hanging, jumping and rearing) in the h preceding mealtime compared to controls after two weeks of PM treatment at ZT 9 (Fig 1D and Fig. 2A–C). Those mice fed PB began to show a pre-prandial increase in high activity by day 10 of PM as compared to controls (Fig. 1D and Fig. 2A). Similarly, examination of individual behaviors for HF mice show statistically significant pre-prandial increases in the fraction of time spent searching in the food bin and hanging cuddled (upside down) compared to controls (Fig 1E–F and Fig. 2B–C, respectively). It is worth noting that PMs caused a noticeable dampening in the nighttime activity of mice in HF and PB groups (see ZT 18–20 in Fig 1D). Also, the nighttime activity peak occurred later than normal in the dark cycle, possibly as the result of the extensive handling that these mice underwent from being video recorded every 2–3 days.

To account for differences in total activity between groups, and between mice within groups, the seconds of activity in the 2.5 h preceding mealtime were divided by the total seconds of activity in the 23.5–24 h recording period on an individual basis. Mice in the HF group show an earlier onset of FAA than mice in the PB group, as measured by their significant pre-prandial increase in fraction of time of high activity (p<0.01 for day 7, 10, 13 and p<0.05 for day 15). Significant FAA for HF treated mice subsides for this group after day 15, which contrasts with our initial published report that suggests that HF entrainment is stably increased from days 14 to day 28 (measured weekly) [13]. In this experiment we observed only a trend toward increased activity in measurements of high activity until day 15 (Fig. 2A). Mice in the PB group showed an increase in the fraction of high activity that becomes evident by day 10 and sustained this increase in high activity on days 13, 15, and 20 (Fig. 2A). Examination of selected individual behavior measurements, particularly those of food bin entry and hanging behaviors, showed similar trends as high activity. HF and PB-treated mice showed an increase in searching in the food bin by day 7 for both groups (Fig. 2B). Additionally, HF mice showed a strong increase in hanging cuddled from days 4 through 15 while PB treated mice only showed a significant increase in hanging cuddled on days 10 and 17 (Fig 2C).

To express the relative amount of anticipatory activity per individual mouse, we divided the maximum number of seconds per one hour bin of high activity in the 3 h preceding mealtime by the maximum number of seconds of high activity during the dark cycle (Fig. 2D). By this definition, some mice in the AL control group show single bouts of high activity comparable to the night peak, but this behavior pattern is intermittent and largely inconsistent, occurring in different animals and disparate across time points. On the other hand, many of the mice in the PB and HF groups showed notable (i.e. yellow to red) high activity ratios from days 7 to 15 of the experiment (Fig 2D). Consistent with our previous report [13], some PM mice do not show increased activity preceding the meal consistently; for examples, see PB 569, PB 581, HF 566, and HF 576. It should be noted that the mice that do not consistently anticipate PMs almost never show high levels of anticipatory activity and show only the lowest activity ratios (indicated by yellow coloring).

### Experiment 2: Temperature, Behavioral, and Immediate Early Gene Responses to Palatable Meals of High Fat and Cheese

**Figure 3:**
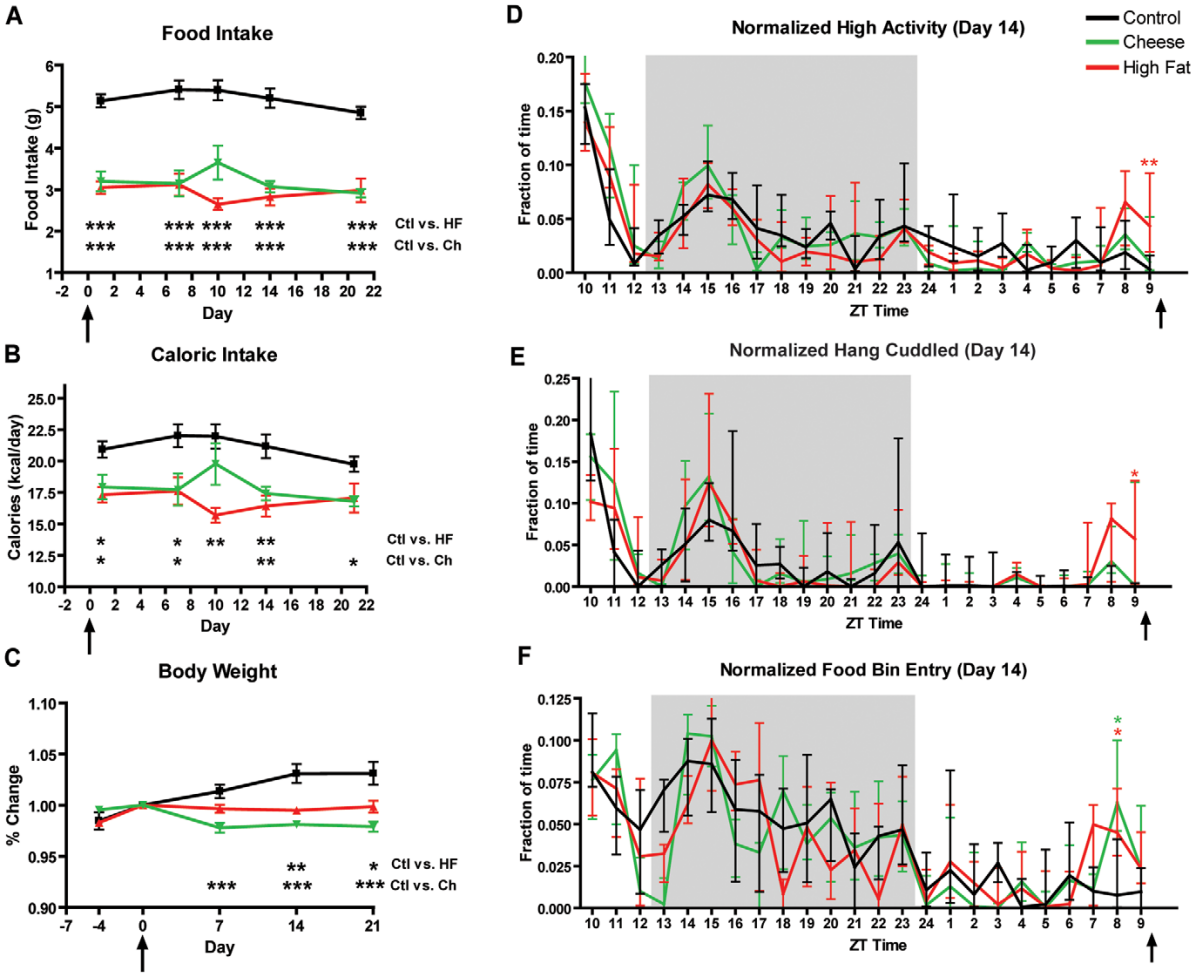
Food intake, caloric intake, change in body weight, and activity profiles of mice in experiment 2. (A) Male mice fed high fat diet and cheese show a significant decrease in chow intake compared to mice with ad libitum access. (B) Male mice fed cheese and those fed high fat show a significant decrease in total caloric intake (sum of calories from chow plus and PM) in comparison to mice with ad libitum access. Caloric intake values were estimated from nutritional facts provided by the respective companies. (C) Male mice fed high fat keep a stable body weight, while mice fed cheese show a modest but statistically significant decrease in body weight after 21 days of PM access. AL controls show a gradual increase of body weight. Change in body weight is calculated by normalizing all body weights to the their respective value at day 0 (first day of PM presentation) (D) Fraction of time per hour spent walking, hanging, jumping, or rearing observed during each hour of the 24 hour recording of Day 14 of experiment 2. Mice fed high fat diet show statistically significant increase in high activity at ZT 9 (E) Fraction of time per hour spent sniffing food bin during 24-hour recording of Day 14. Both HF and CH groups show a statistically significant increase in food bin entry at ZT 8 (F) Fraction of time per hour spent hang cuddling during the 24-hour recording of Day 14. Mice fed high fat show an evident and statistically significant increase in hang cuddling at ZT 9. Arrows after ZT 9 below the y-axis indicate the time of PM presentation. For 1A thru 1C, bars represent means +/− SEM. Statistical comparisons were performed using an ANOVA followed by Tukey-Kramer multiple comparisons test. For 1D thru 1F, bars represent medians ± IQ Range (significance was tested with Mann-Whitney test with Dunn’s post test. * denotes p<0.05, ** denotes p<0.01.

Next we performed a study of the behavior, body temperature, and c-Fos induction in response to daily timed PMs of HF and cheese (CH), a novel fatty meal that we hoped would induce FAA at least as well as PB. Consistent with our previous findings, PM-treated mice consumed significantly less chow than controls by their first day of scheduled feeding (p<0.001 for all days, Fig. 3A) and consumed all treats within 30 min of food presentation. The amount of calories from both PMs corresponded to 30–35% of the total daily caloric intake. Mice fed PMs also have a reduced caloric intake that is sustained throughout the experiment (Fig. 3B). Unlike the mice in experiment 1, PM-treated mice show a decrease or a lack of an increase in body weight (Fig 3C). AL mice show an increase in body weight across the 21 days of the experiment (p = 0.005; ANOVA on slope of least-squares linear fit of weight versus day for each mouse), while CH mice show a modest decrease in body weight during this period (p = 0.02), and HF mice do not show a significant change (p = 0.73). The difference in body weight between control and CH-treated mice is significantly different from day 7 onwards (Fig 3C). Overall, these changes in body weight are small: on day 21, CH mice have a 4% decrease in body weight while AL controls show a 3% increase. In future studies it would be of interest to test the long-term effects of PMs on body weight homeostasis and health in mice.

Mice receiving daily timed HF, CH, or handled as a control (“AL” group) were video recorded to measure activity on days −4, 0, 7, 10, 14, and 21. Mice receiving daily HF showed a significant increase (p<0.01) in the fraction of high activity at ZT 9 compared to the AL control group on the 14^th^ day of treatment (Fig. 3D). Similarly, mice in the HF group displayed a significant preprandial increase (p<0.05) in the fraction time spent hanging cuddled on this day of the experiment at ZT 9 (Fig. 3F). However, the CH treated mice did not show evident anticipatory activity on day 14 (Fig. 3D) or hanging (Fig. 3F) when compared with controls. Interestingly, on day 14 both CH and HF mice spent double the fraction of time sniffing the food bin preceding meal time at ZT 8 compared to the AL controls (Fig. 3E).

**Figure 4:**
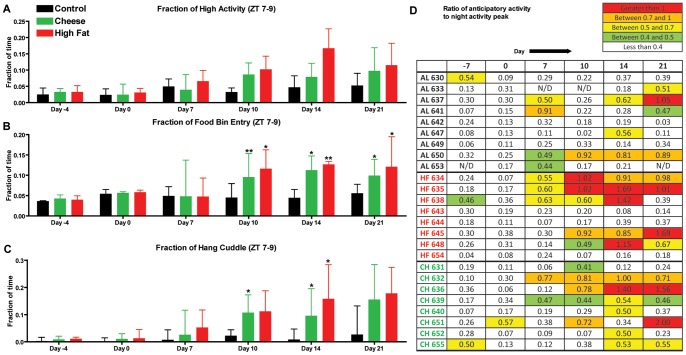
Home cage activity of mice in Experiment 2. (A) Fraction of time spent on high activity behaviors during the 2.5 h period preceding PM presentation (The sum of time of high activity behaviors during the 2.5 h before PM is divided by total seconds of high activity behaviors during entire recording period). Fraction of time spent sniffing the food bin (B), and hang cuddling (C) during the 2.5 h period preceding PM presentation. (D) The maximum number of seconds per hour of high activity behaviors in 2.5 h preceding mealtime is divided by the maximum number of seconds of high activity at nighttime. Ratios greater than 1 are shown in red, between 0.7 and 1 in orange, between 0.5 and 0.7 in yellow, and between 0.4 and 0.5 in green. Bars represent medians +/− IQR. Statistics were performed using Mann-Whitney Test * denotes p<0.05 and ** denotes p<0.01.

In contrast to the PM mice in experiment 1, PM-treated mice in experiment 2 had similar amplitudes of nighttime activity peaks when compared to controls (Fig. 3D). To normalize the data across each treatment, we computed the fraction of pre-prandial high activity (ZT 7–9) relative to total high activity. Mice in the HF and PB groups showed trends toward– increased high activity but none of these values reached statistical significance (Fig. 4A). Food bin entry behavior was stably and significantly elevated from day 10 onward in both CH- and HF-treated mice (Fig. 4B). This suggests that the mice were not showing the full manifestations of FAA in terms of increasing high activity behaviors but were aroused and examining the food bin for a PM. In terms of hanging cuddled, the most sensitive single measure of FAA [15], HF mice showed a trend toward increased fraction of time spent hanging, which becomes significant on day 14 (Fig. 4C). For CH-treated mice, the fraction of time spent hanging increases gradually and becomes statistically significant on days 10 and 14 (Fig. 4C).

To account for individual variations in anticipatory activity, the maximum number of seconds of high activity in the 3 h preceding mealtime was divided by the maximum number of seconds of high activity during the dark cycle (Fig. 4D). Occasionally, mice in the AL control group showed single bouts of high activity comparable to the night peak, but generally this behavior pattern is not consistent (with the notable exceptions of AL 637 and AL650). On the other hand, many of the mice in the HF and CH groups showed consistent and sustained high activity after 7 to10 days of PM treatment: mice HF 634, HF 635, HF 636 HF 645, HF 654, CH 632, CH 636, CH 639, and CH 651 showed FAA throughout the experiment (Fig. 4D). Moreover, those PM-treated mice that do not show high preprandial activity ratios were fairly consistent (see HF 643, HF644, HF 654, CH 631, CH 640, and CH 652) and represent the fraction of “non-responders” that occurred in our previous PM experiments [13].

**Figure 5:**
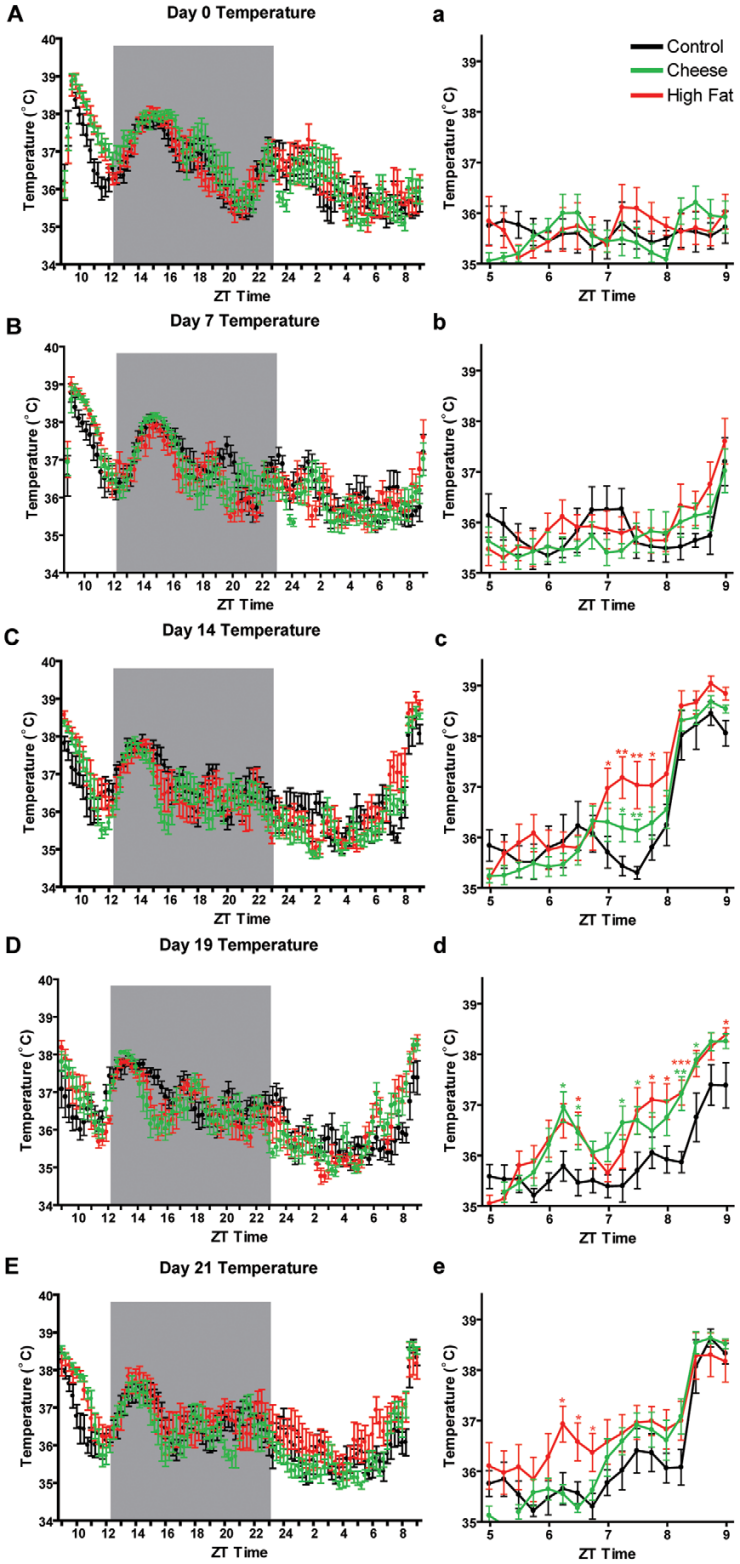
Body temperature profiles of mice in Experiment 2. Mean temperatures at first day of PM presentation, day 0 (A), day 7 (B), day 14 (C), day 19 (D), and day 21 (E). Bars correspond to IQ ranges. Statistical comparisons were performed using Mann-Whitney test; * denotes p<0.05, ** denotes p<0.01.

**Figure 6:**
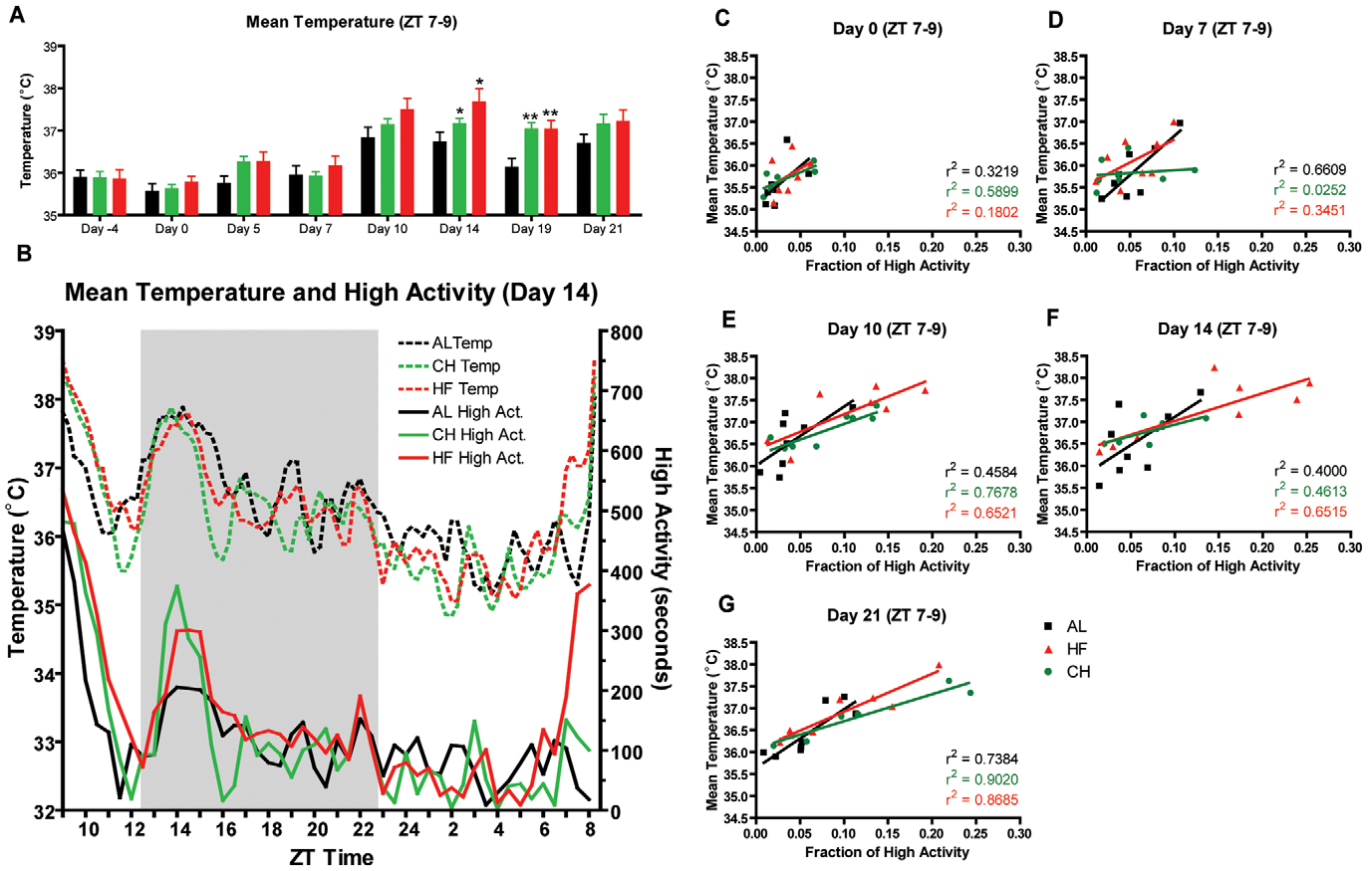
Body temperature is moderately correlated with Food Anticipatory Activity. (A) Mean temperature during the 2.5 h preceding PM presentation. Error bars represent SEM. Statistical comparisons were performed using an ANOVA followed by Tukey-Kramer multiple comparisons test, * denotes p<0.05, ** denotes p<0.01, and *** denotes p<0.001. (B) Mean temperature and high activity 14 days after start of PM feeding regimen. Mean temperature is represented by dashed lines in top half of the panel (corresponding to left y-axis) and mean high activity in seconds is represented by the solid line in the bottom half of the panel (corresponding to right y-axis). Temperature plotted against the fraction of high activity during the 2.5 H preceding mealtime for day 0 (C), day 7 (D), day 14 (E), and Day 21 (F). Linear regression lines and R^2^ are shown.

In this experiment, we also measured body temperature using an implanted device that recorded temperature every 15 minutes for the duration of the experiment. Measurement of core body temperature in mice handled as a control, fed cheese, or fed high fat all had similar distributions on the first day of measurement, day 0 (Fig. 5). There was a notably high temperature for all groups of mice at ZT 9–10, presumably due to having stressed the mice by re-caging and transporting them to the video recording room. After 7 days of PM treatment, mice fed CH or HF showed a marked preprandial increase in body temperature at ZT 8 (Fig. 5B). By day 14, the control group also showed a marked increase in body temperature at ZT 8 and this can be attributed to daily handling, which can entrain body temperature [16] (Fig. 6C). The mice fed CH or HF showed an increase in body temperature preceding that of controls by about one hour, starting at ZT 7 their body temperatures were significantly higher than that of controls but by ZT 8 there was no difference in mean body temperature (Figure 6c). On day 19, a time point when there had been less disturbance of the mice as they had not been video recorded during the prior 4 days, the preprandial increase in body temperature for mice fed CH and HF quite evident beginning at ZT 6 and remains increased over that of AL control mice for most of the measurements prior to ZT 9 when PMs were delivered (Figure 5D). On day 21 of the experiment, when mice had been re-caged and transported to the video recording room, we observe that the preprandial increase in body temperature was much less pronounced in PM treated mice; CH-treated mice do not show a significantly higher mean body temperature than controls and the HF-treated mice were only significantly increased at a few measurements during ZT 6–7 (Fig. 5E). This suggests that there is a masking effect of handling the mice that makes it difficult to observe temperature entrainment to PMs on the background of entrainment to handling. We plotted preprandial mean temperature in a 2 h bin preceding meal time, noting that the mean temperature of PM-treated mice was significantly elevated on days 14 and 19 only (Fig. 6A). Since we recorded temperature continuously and behavior twice per week this allowed us to examine both variables simultaneously (Fig. 6B). The mean body temperature and preprandial activity appeared to be positively correlated (for example see HF mice Fig. 6B). To that end, we plotted linear regressions for mean temperature and the fraction of high activity in the 2 h preceding expected meal time at days 0, 7, 10, and 14, obtaining r^2^ values that appeared to increase over the course of the experiment for all groups of mice (Fig. 6C–G). None of the differences in correlation values between groups at a given time point were statistically significant (all had a p value of at least 0.15 in two-tailed tests for differences based on nonparametric bootstrap re-sampling of temperature/activity pairs). We also tested for significant changes in the increase in correlation values within groups over time. The only comparison with two-tailed p<0.05 was between day 7 and 21 for CH mice (p = 0.007; bootstrap re-sampling), but note that this increase in correlation between temperature and activity may be due in part to increased variance of each at day 21, as the residual variance of the regression fit did not decrease significantly (p = 0.34; bootstrap re-sampling).

**Figure 7:**
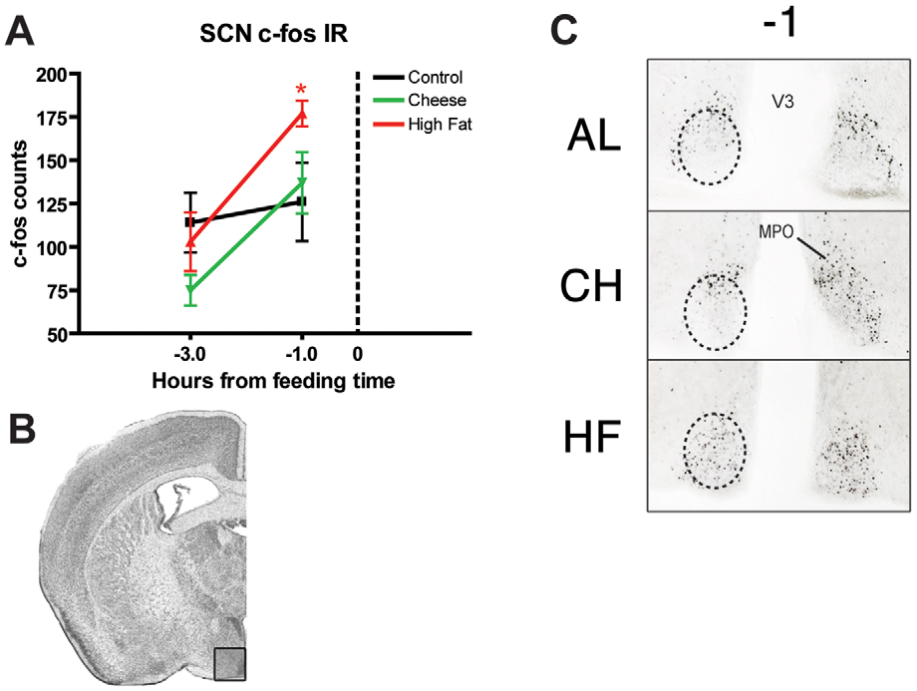
c-Fos expression in the Suprachiasmatic Nucleus (SCN) of mice fed rodent high fat diet and cheese. (A) Graph showing mean (±SEM) number of c-Fos-immunoreactive (cfos-IR) nuclei in the SCN of AL controls (black), CH (green), and HF (red) at ZT 5 (TP -3 hours) and ZT 7 (TP -1 hours). TP 0 represents time of PM presentation as highlighted by the vertical dotted line on the x-axis. (B) Representative coronal brain atlas image with Nissl stain showing location of the region examined (adapted from Paxinos and Franklin). (C) Representative micrographs at TP -1 of c-Fos expression in the SCN. MPO, medial preoptic area: V3, third ventricle.

**Figure 8:**
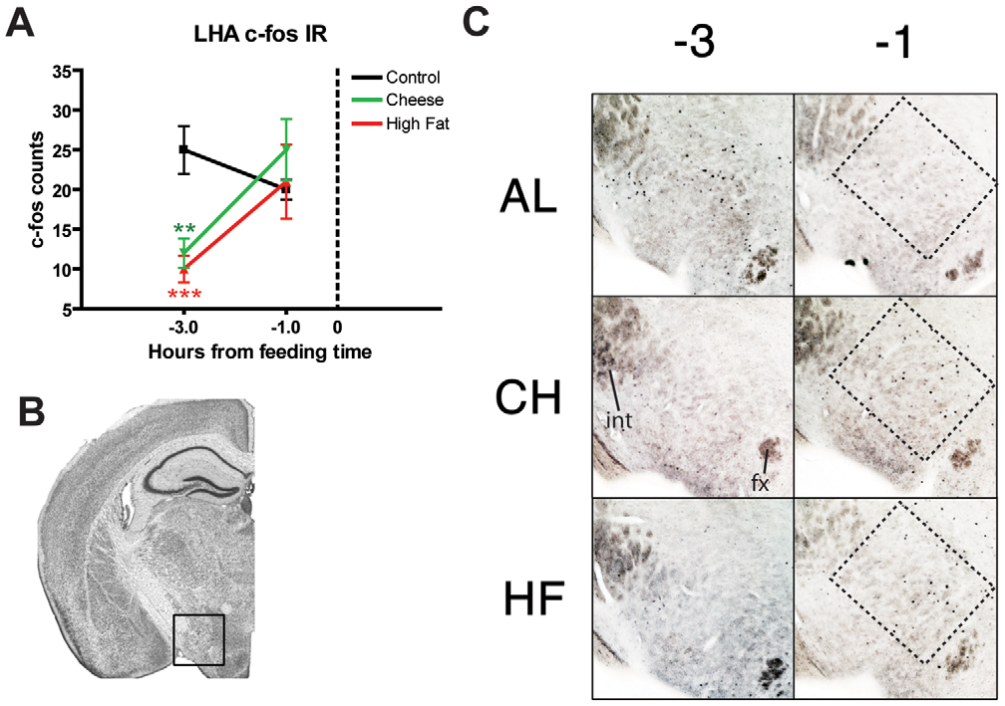
c-Fos expression in the Lateral Hypothalamic Area (LHA) in mice fed rodent high fat diet and cheese. (A) Graph showing mean (+/− SEM) number of c-Fos-immunoreactive (c-Fos IR) nuclei in the LHA of AL controls (black), CH (green), and HF (red) at ZT 5 (TP -3 hours) and ZT 7 (TP -1 hours). TP 0 represents time of PM presentation as highlighted by the vertical dotted line on the x-axis. CH and HF mice show a lower and statistically significant c-Fos count compared to AL controls. Counts for CH and HF reach similar values to AL group by TP -1. (B) Representative coronal brain atlas image with Nissl stain showing location of the region studied (adapted from Paxinos and Franklin). (C) Representative micrographs at TP -3 and -1 of c-Fos expression in the LHA. int (internal capsule); fx, fornix.

**Figure 9:**
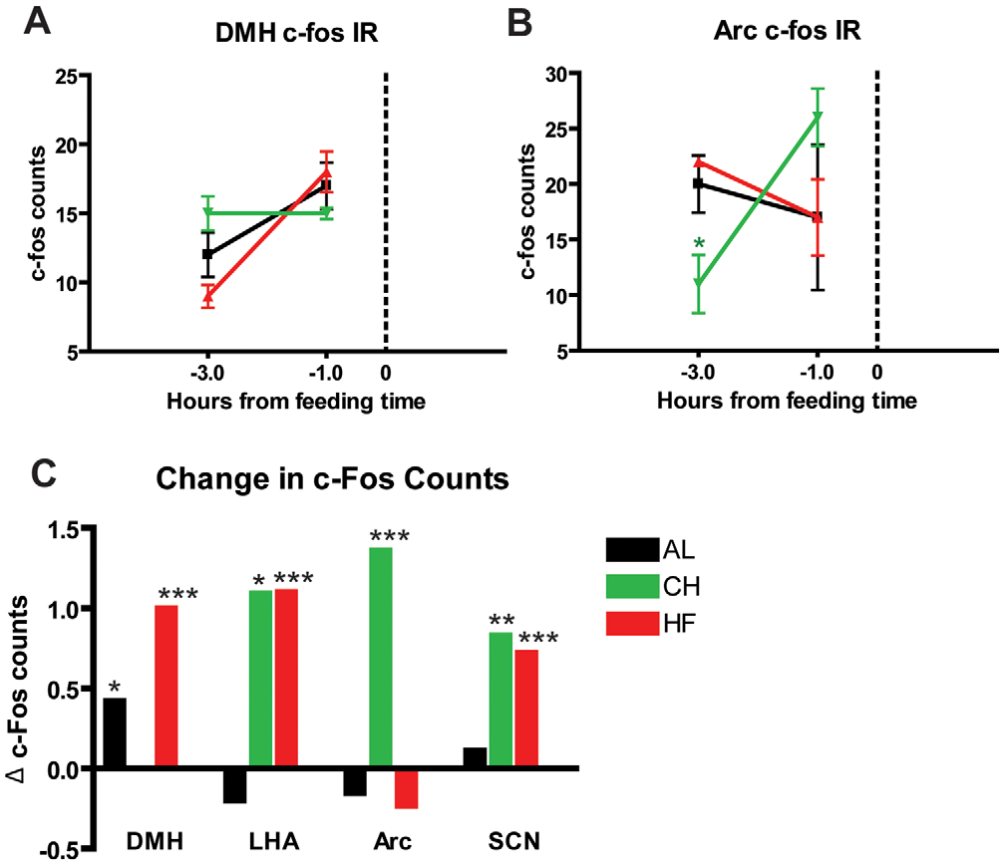
Fold change in c-Fos immunoreactive counts hypothalamic nuclei of mice fed rodent high fat diet and cheese. The Dorsomedial Hypothalamus (DMH) and Arcuate Nucleus (Arc) (Panels A, and B respectively) of AL controls and PM-fed HF and CH groups at TP -3 (ZT 5) and TP -1 (ZT 7). (C) The change in c-Fos counts from TP -3 to TP -1 show an positive increase in c-Fos counts in HF mice for the DMH, LHA, and the SCN, and a positive increase in counts of CH mice for LHA, Arc, and SCN.

To determine whether there was entrainment of neural activity in mice on PM feeding schedules, cohorts of mice were culled at ZT 8 (1 hour prior to meal time, termed time point (TP -1)) and ZT 6, 3 hours prior to meal time (“TP -3”) to determine the changes in hypothalamic c-Fos expression in this timeframe when FAA induction is more evident (at least n = 4 for each treatment and time point) (Figs. 7–9). In the SCN, c-Fos expression was significantly increased (p<0.05) in HF groups at TP -1 compared to AL and CH counterparts, with mean counts of ∼175 and ∼125, respectively (Figure 7A). Mice in the CH group do not show higher c-Fos counts at TP -1 compared to controls, but do show a similar trend in the changes of c-Fos counts between TP -3 and TP -1: with both HF and CH groups showing a significant ∼75% increase in c-Fos counts from TP -3 to TP -1 (Figure 9C, p<0.001 and p<0.01 respectively). Finally AL controls showed a modest but non-significant increase in c-Fos expression. In the lateral hypothalamic area (LHA), c-Fos expression is significantly reduced in CH and HF groups at TP -3 (p<0.01 and p<0.001 respectively) compared to AL controls (Figure 8A). However, c-Fos counts in all groups (even AL) converge to ∼25 counts, resulting in increases in c-Fos expression of ∼100 percent for HF and CH groups (p<0.001 and p<0.05 respectively) and a modest decrease of around 20 percent in AL controls (Figure 9C). In the dorsomedial hypothalamus (DMH) we observed similar counts converging to similar values at TP -1 (∼15 counts), resulting in positive changes in c-Fos expression for AL and HF groups (p<0.05 and p<0.001 respectively) and no change for CH groups Figure (9A and 9C). Finally, counts in the arcuate nucleus (Arc) show a decreased c-Fos expression in CH groups at TP -3 (p<0.05) compared to AL controls, while HF groups show similar counts to ALs in both TP -3 and TP -1 (Figure 9B). Despite the greater variability in c-Fos counts at TP -1, calculations of delta c-Fos show a significant ∼150 percent increase in the expression of c-Fos in CH groups (p<0.001) with a modest decrease of ∼20 percent in AL and HF groups (Figure 9C).

### Experiment 3: 129S1 inbred mice do not show anticipatory activity for daily, timed high fat diet meals

Given that behavioral anticipation was not fully penetrant in the C57BL6/J strain, we tested an alternative inbred strain of mice commonly used in transgenic experiments, the 129S1 strain. An additional motivation for the study was our observation in a pilot study that 129S1 mice on a 60% CR regimen redistribute almost all of their high activity behaviors to the hours preceding feeding time and do not retain a night-time activity peak. We hypothesized that scheduled feedings of PMs would elicit an analogous response and yield a more robust FAA signal compared to the studies in C57BL/6J mice.

**Figure 10:**
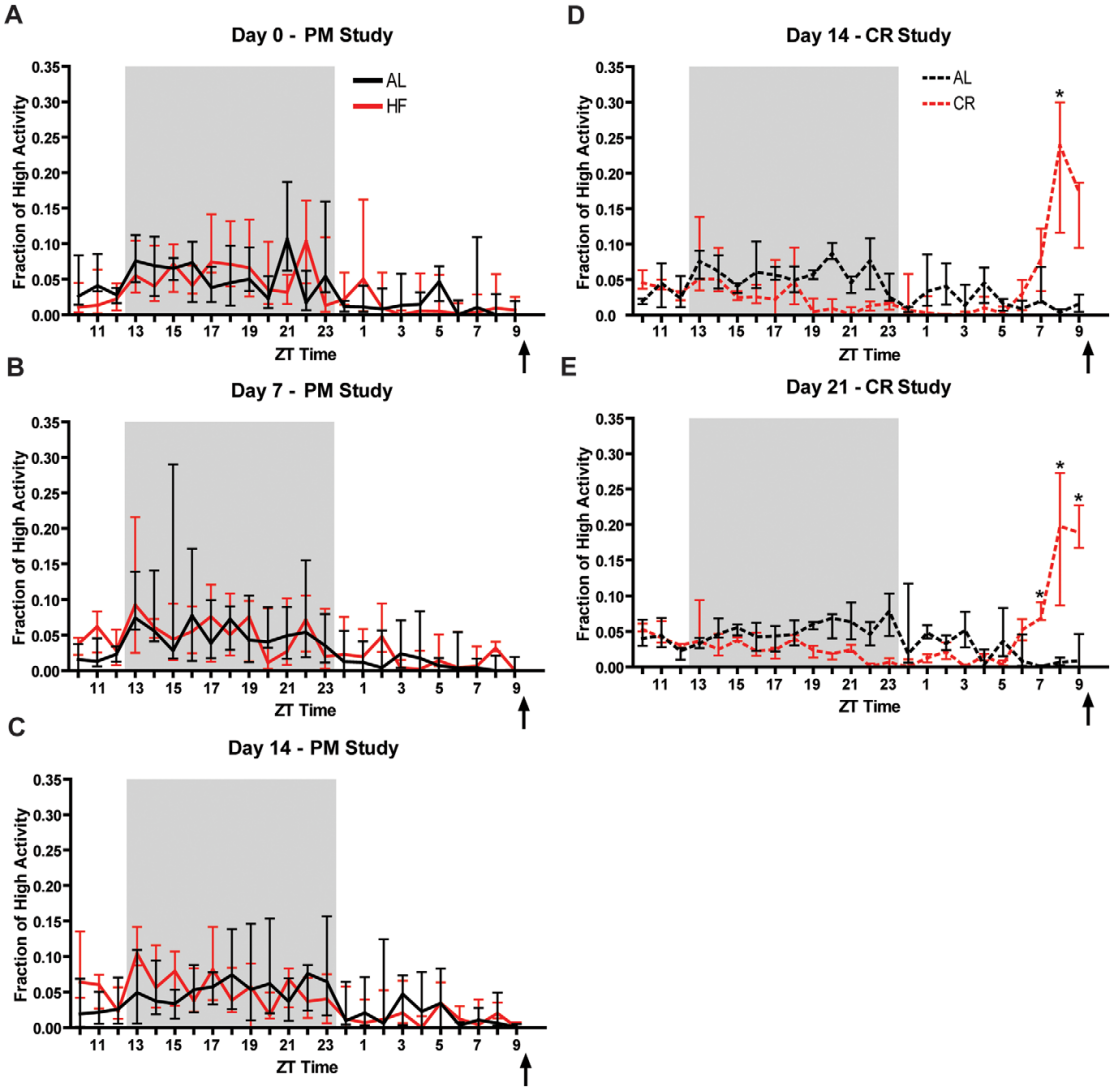
Daily palatable meals of rodent high fat diet do not cause a pre-prandial increase in activity in 129S1 mice, which do show robust food anticipatory activity for 60% CR (Experiment **3).** Fraction of time per hour spent walking, hanging, jumping, or rearing observed in PM-fed 129S1 mice during each hour of the 24-hour recording in Days 0, 7, and 14 (A–C). Free-feeding mice fed high fat diet at ZT 9 do not show any significant differences in pre-prandial high activity after 14 days in feeding schedule. Fraction of time per hour spent walking, hanging, jumping, or rearing observed in 129S1 mice in 60% CR schedule during each hour of the 24-hour recording in Days 14 and 21 (D–E). Mice subject to calorie restriction show significantly higher pre-prandial high activity in Days 14 and 21. Lines represent medians +/− IQ Range (significance was tested with Mann-Whitney test with Dunn’s post test. * denotes p<0.05.

When placed on HF PM feeding schedule, 129S1 mice fail to show an increase in the fraction of high activity preceding mealtime at any time point measured (Days −7, 0, 7, 10, and 14). Even after 14 days, there is no indication of FAA or an evident redistribution of nighttime activity compared to controls despite the fact that the mice consumed the HF treat avidly (Fig. 10A–C). By contrast, 129S1 mice fed a 60% CR diet once per day at ZT 7 showed an evident increase in the fraction of pre-prandial high activity. For example, days 14 and 21 of 60% CR are plotted in Figure 10D–E. Finally, it is notable that under our conditions 129S1 mice do not have a strong peak of dark cycle activity during PM-treatment, and this lack of nighttime activity is even more apparent under a CR feeding schedule (Figure 10D–E).

## Discussion

In this study we show that daily, timed feeding of fat-rich PMs induces moderate FAA, increases preprandial body temperature, and entrains c-Fos activation signatures in several hypothalamic nuclei. Despite the avid consumption of the palatable snacks across all groups, FAA in PM-treated mice was moderate: never exceeding 3 times the values of AL controls in terms of group data and with some individuals consistently failing to develop any FAA. In experiment 2 we attempted to minimize handlings/video recordings of all mice in order to bring down ‘background’ activity and entrainment in AL controls. Despite our efforts to reduce handling, it seems that the mere delivery of a control chow pellet to AL mice was enough to elicit bursts of anticipatory activity in a few mice in the group by day 7 (Fig. 4D). The physiological entrainment to daily disturbance as an entraining stimulus is particularly evident when examining body temperature, which shows a small preprandial increase in AL control groups by day 14 and a very clear increase by day 21. In fact, when comparing with pre-prandial mean temperature and FAA there are several control mice that show correlations comparable to that achieved by several of the HFD or CH entrained mice (Figure 6G). The entrainment of temperature due to handling has been well documented [16], but the entrainment of behavioral anticipatory activity to handling is generally not achieved easily [17]. For example, in previous work we scheduled 2 h of mid-day running wheel access for C57BL/6J male mice and did not observe any anticipatory activity across the 21 days of the experiment, despite active wheel running by all mice daily [18]. Examination of the behavior of individual PM-treated mice shows that some animals express consistent anticipatory activity (Figure 2D, 4D). Both activity matrices (Figure 2D and 4D) and temperature data (Figure 6) suggest several strategies to optimize the use of the PM paradigm in the study of FAA: (1) the handling of mice needs to be minimized, perhaps by using automatic feeding devices, as an attempt to minimize the baseline activity of AL mice and (2) the signal of PM FAA needs to be increased, possibly by finding better entraining meals, or (3) comparing activity within a mouse pre-and post-treatment rather than using separate AL control mice.

The neural substrate for FAA is still a matter a contention as both evidence for and against structures such as the dorsal medial hypothalamus or the nucleus accumbens has been demonstrated [11], [19], [20], [21], [22], [23]. In addition, several peptides and hormones have been proposed to impact the putative FEO, including ghrelin [24]. Studies by Angeles-Castellanos *et al*. have shown the ability of daily-timed PMs to entrain circadian-like oscillations of period proteins in corticolimbic brain structures [12], [15], [25]. This PM paradigm, however, failed to affect the per1 and c-Fos oscillatory patterns in the SCN, probably due to the entrainment of these rats to a regular light-dark (LD) cycle. This differential activation of certain brain areas, even in the presence of light cues, underscores the ability of the PM paradigm to entrain circadian oscillatory patterns in these rodents without necessarily acting through the SCN. This, however, does not preclude an interaction between the SCN and the putative FEO, as indicated in PM experiments conducted constant darkness where per1 expression was affected in the SCN and the periventricular nucleus of the thalamus (PVT) [26]. These results are at odds with two immunohistochemical studies that suggest that Per2 rhythm is unaffected by daily timed PM in the motivational and emotional areas of the brain, despite the fact that all rodents consumed these treats avidly [27], [28]. These discrepancies are likely due to the differences in the length or design of the study but also underscore the point that there is little lab-to-lab agreement with respect to findings in the FAA field [15]. Given the possibility of a long latency in PM-induced FAA and unclear IHC data, we sought to extend feeding schedules to at least 21 days, and use a more general indicator of neuronal activation for our immounohistochemical studies, the immediate early gene c-Fos. We observed significant entrainment of c-Fos staining in several hypothalamic structures when comparing changes in counts in the 3 h preceding meal presentation. Future studies will examine other hypothalamic and corticolimbic structures in a broader range of time and use other indicators of neuronal activity/phase.

The differential response of the 129S1 strain to PM and CR feeding schedules suggests the presence of a food-deprivation oscillator that is dissociable from a reward-expectancy oscillator. However, more PM meal experiments conducted on the 129S1 strain are needed to confirm the validity of this claim. This would be consistent with the hypothesis regarding the presence of a distributed system of circadian oscillators that are entrained by unique zeitgebers. In fact, known circadian oscillators are constructed so as to be respond to a particular stimulus and resist entrainment by others [29]. For example, the circadian rhythm of an explanted SCN is immune to alterations in external temperature whereas other tissues are entrained by shifts in external temperature [30]. One similarity between PM and CR/RF meal anticipation is that core body temperature increases preceding the meal (Figure 6A) [15], [20]. However, any causality between increased body temperature and FAA induction remains unproven [23], [31], [32], [33], [34]. In fact, in our recent study of feeding CR meals at 4 hour intervals, we observed a clear dissociation of body temperature and FAA, suggesting that these can be entrained independently and one is not required for the other to occur [35].

Interestingly, the 129S1 inbred strain showed no anticipatory activity for daily HFD but had robust FAA for a timed 60% CR meal (Figure 10). As many knockout mice are derived at least in part from the 129 lineage, we thought it worth reporting that this strain background is not suitable for PM anticipation studies. The 129 mice consumed the HFD quite rapidly, arguing against a sufficient lack of palatability of this meal. One possible explanation for the differential induction of FAA by CR but not by PM schedules could be the differences in brain anatomy reported in these strains. In particular, 129S1 mice manifest agenesis and dysplasia of the corpus callosum [36], [37], [38]. It is possible that one of the many anatomical abnormalities of the 129S1 line include an impaired ability to time palatable rewards but not 60% CR meal time. It is also notable that in prior studies female C57BL/6J mice failed to show FAA for a HFD. As female C57BL/6J females have no deficit in timing 60% CR meals a comparative study in C57BL/6J males versus females may also be informative for PM anticipation circuitry. Such studies could dissociate a PM anticipation circuit modulated by motivational drive from a caloric intake sensing circuit involved in homeostatic control [39]. Further investigation of anticipatory behavior in either the 129S1 strain or female C57BL/6J mice may open up avenues for using mouse genetics, sex hormones, and/or c-Fos studies to tease apart the neural systems governing FAA.

## Materials and Methods

### Ethics Statement

These experiments were approved by the Caltech Institutional Animal Care Committee under protocol #1567. Every effort was made to minimize pain, distress, and the overall number of animals used in this study.

### Mouse Strains and husbandry

Twelve-week old male C57BL/6J and 129S1 mice were purchased from the Jackson Laboratory and single housed for at least 3 days prior to initiating the experiment. Mice had *ad libitum* access to Rodent Diet 5001 and water throughout the experiment in the Caltech Animal Facility at 13∶11 LD cycle and a temperature between 22–24 Celsius. Mice were housed in clear polypropylene cages next to each other during housing and video recordings. Prior to each video recording, mice were weighed and placed in recording cages (with slightly less bedding and no cotton nestlet), and transported on a cart to the recording room within the same animal facility.

### Behavioral and Body Temperature Measurements

Mice in experiment #1 were video recorded for 23.5 to 24 h on days −4, −2 0, 4, 7, 10, 13, 15, 17, and 20 while those used in experiment #2 were video recorded on days −4, 0, 7, 10, 14, and 21. For all experiments, video recordings are started as soon as the mice are placed on their recording racks and given their palatable treat or control pellet. Following the video recordings, the mice are transported back to the husbandry room, recaged, and provided with their respective meal. Video recordings were analyzed with HomeCageScan 3.0 (Cleversys, Inc.) [14], [40], [41]. Behaviors were defined as described previously [13], [15], [18]; for the purposes of our analysis we examined only the behaviors of hanging vertically, hanging cuddled (upside down), jumping, walking, and rearing up and down. These behaviors were summed together to measure time engaged in “high activity” behaviors [13], [14]. All other behaviors, such as drinking, resting, grooming, etc were ignored. Data were exported into 24 one-hour bins (or 30 minute bins for Fig. 6B). Data were normalized by dividing the number of seconds per hour of a particular behavior (e.g., hanging cuddled, food bin entry, or high activity) by the total number of seconds engaged in that behavior across the ∼24 hour video recording. Dim red lighting was provided during the 11 hr dark cycle with red LED lights (LEDwholesalers.com) to allow acceptable contrast when recording during the night cycle. Behavior data was evaluated for statistical significance using non-parametric tests as indicated using GraphPad InStat.

Experiment 1 was done in two independent rounds of n = 4 mice per group, yielding n = 8 per group in total. At the start of “day 0”, mice were split into three groups: (1) AL controls receiving a 5001 chow pellet as a disturbance control (2) HF group receiving a daily meal of 0.9 g of Bio-SERV High fat Diet (containing 35.5% fat, 20.0% protein, and 36.3% carbohydrates by weight, (3) PB group receiving a daily portion of 0.75 g Skippy® brand peanut butter. The procedure was repeated daily for 20 days, with mice receiving daily treats or disturbance control pellets at ZT10 each day. Mice in experiment 1 were kept in recording cages throughout the duration of the experiment. Given their reduced amount of bedding, mice were provided fresh recording cages every two days to ensure appropriate hygiene. Body weights and food intake for all mice were calculated during the cage changes to minimize the amount of handling.

For temperature monitoring experiments (“experiment 2”), 25 C57BL/6J male mice were single housed for at least 3 days and surgically implanted with iButtons [15] [42] (Maxim Integrated Products) programmed to record core body temperature at discrete 15-minute intervals to the nearest 0.0625 degree Celsius. Post-operative monitoring and recovery time was provided for at least seven days before putting mice on PM or control feeding schedules. Mice were split into three groups: (1) AL controls receiving a 5001 chow pellet as a disturbance control, (2) HF group receiving a daily meal of 0.9 g of Bio-SERV High fat Diet, and (3) CH group receiving a daily treat of 0.6 g Havarti cheese (purchased from Fresh and Easy© market). Unlike experiment 1, mice were housed in regular cages (with standard amount of bedding) and cage changed the day of the recording to minimize disturbance or any sudden changes in temperature due to handling. Food intakes and body weights were recorded before weekly video recordings.

For experiments on the 129S1 line, mice were video recorded for 23.5 to 24 h on days -7, 0, 7, 14 and 21. Experiment 3 was done in independent rounds of CR and PM feeding schedule with n = 4 and n = 6 per group respectively. Mice on the CR study were split into an AL and CR group at the start of “day 0”. Mice on CR received a single pellet that corresponded to 60% of their AL food intake (as calculated during the week preceding the start of feeding schedules) at ZT9 each day. The procedure was repeated daily for 21 days with mice receiving 60% CR or disturbance control pellets at ZT9 each day. PM meal feeding schedules were started on a different cohort of 129S1 mice on day 0, with AL controls receiving a 5001 chow disturbance control pellet, and HF mice receiving a single daily meal of 0.75 g of Bio-SERV High Fat Diet corresponding to 30–35% of their daily caloric intake. In this experiment, HF servings were lower than those of experiment 2 to reflect the reduced number of calories consumed by these mice. This procedure was repeated daily for mice on the PM study for 21 days with mice receiving single AL disturbance control pellet or a daily HF meal at ZT 9.

### c-Fos activation studies

The brains were removed from mice over-dosed with avertin anesthetic and immersion-fixed in 10% buffered formalin (Sigma) for at least 24 h and sent to NeuroScience Associates (Knoxville, TN). Upon receipt of the brain tissue, they were treated with 20% glycerol and 2% dimethylsulfoxide to prevent freeze-artifacts. They were then multiply embedded with up to 25 brains per block in a gelatin matrix using MultiBrain Technology™, which enables simultaneous sectioning of the entire block of brains. The block of embedded tissue was allowed to cure and then was rapidly frozen by immersion in isopentane chilled to −70°C with crushed dry ice. Blocks were mounted on a freezing stage of an AO 860 sliding microtome and sectioned coronally at 35 µ thickness. All sections cut were collected sequentially into a 4×6 array of containers filled with ‘antigen preserve’ (buffered ethylene glycol).

For immunochemistry, the sections were stained free-floating. All incubation solutions from the blocking serum use Tris buffered saline (TBS) with Triton×100 (TX) as the vehicle; all rinses are with TBS. After a hydrogen peroxide treatment and blocking serum, the sections were immunostained with a primary anti-c-Fos (Novis, rabbit anti c-Fos) 1∶10,000 antibody overnight at room temperature. Vehicle solution contains 0.3% TritonX-100 for permeabilization. To visualize the location of binding site of the primary antibody an avidin-biotin-HRP complex (details in Vectastain elite ABC kit, Vector, Burlingame, CA) is applied. After rinses, the sections were treated with diaminobenzidine tetrahydrochloride (DAB) using nickel enchancemnt and hydrogen peroxide to create a visible reaction product and mounted on gelatinized (subbed) glass slides, air dried, dehydrated in alcohols, cleared in xylene and coverslipped.

Fos-stained sections were examined under a Nikon Eclipse TE2000-U light microscope coupled to a computer with NIS-Elements BR 3.0 software. Image saturation was avoided by adjusting exposure time with the brain slice with the highest c-Fos signal. Expression analysis of c-Fos protein was done for the following regions: SCN (0.48 to 0.655 caudal to bregma), LHA (1.255 to 1.755 caudal to bregma), Arc (−1.555 to −1.855 caudal to bregma), and DMH (−1.455 to −1.755 caudal to bregma). Cells immunopositive for c-Fos protein were counted using 10x and 4x lenses. The mean number of fos-positive cells per time point was calculated from individual bilateral counts of three sections from each animal; the biological sample sizes were n = 4–5 per treatment group at two time points. Counts were done automatically by the software after setting an ROI and threshold levels for each brain region, with threshold levels kept constant in between counts of a specific brain region. ROIs were defined in slices based on visible landmarks and comparison with mouse brain atlas of Paxinos and Franklin [43]. Representative images were captured using the CCD camera while doing automated counts. ANOVA followed by Tukey-Kramer post hoc corrections were used to determine statistical differences between groups.
